# A comprehensive bioinformatics analysis on multiple Gene Expression Omnibus datasets of nonalcoholic fatty liver disease and nonalcoholic steatohepatitis

**DOI:** 10.1038/s41598-018-25658-4

**Published:** 2018-05-16

**Authors:** Shanzhou Huang, Chengjun Sun, Yuchen Hou, Yunhua Tang, Zebin Zhu, Zhiheng Zhang, Yixi Zhang, Linhe Wang, Qiang Zhao, Mao-Gen Chen, Zhiyong Guo, Dongping Wang, Weiqiang Ju, Qi Zhou, Linwei Wu, Xiaoshun He

**Affiliations:** 1 Organ Transplant Center, The First Affiliated Hospital, Sun Yat-sen University, Guangzhou, 510080 China; 2 Guangdong Provincial Key Laboratory of Organ Donation and Transplant Immunology, Guangzhou, 510080 China; 3 Guangdong Provincial International Cooperation Base of Science and Technology (Organ Transplantation), Guangzhou, 510080 China; 4 Department of General Surgery, Hui Ya Hospital of The First Affiliated Hospital, Sun Yat-sen University, Huizhou, Guangdong, 516081 China

**Keywords:** Gene expression, Genomics

## Abstract

Fatty liver disease is one of the leading causes of chronic damage in western countries. Approximately 25% of adults in the United States have fatty livers in the absence of excessive alcohol consumption, a condition termed nonalcoholic fatty liver disease (NAFLD). Little is known about the prevalence and genetic background of NAFLD or the factors that determine its development. In this study, we used the Gene-Cloud of Biotechnology Information bioinformatics platform to carry out a comprehensive bioinformatics analysis identifying differentially expressed genes (DEGs), key biological processes and intersecting pathways. We imported 3 Gene Expression Omnibus datasets (GSE66676, GSE49541, and GSE83452). Then, we assessed the expression of the DEGs in clinical samples. We found that CD24 was the only gene co-expressed in all 3 datasets. “Glycolysis/gluconeogenesis”, “p53 signaling pathway” and “glycine, serine and threonine metabolism” were 3 common pathways related to the fatty liver process. In NAFLD tissues, CD24, COL1A1, LUM, THBS2 and EPHA3 were upregulated, and PZP was downregulated. CD24 is a core gene among these DEGs and have not yet been studied of its impact on NAFLD. Co-expressed genes, common biological processes and intersecting pathways identified in the study might play an important role in NAFLD progression. Further studies are needed to elucidate the mechanism of these potential genes and pathways in NAFLD.

## Introduction

Nonalcoholic fatty liver disease (NAFLD) and its subtype nonalcoholic steatohepatitis (NASH) have become an increasingly important clinical and economic burden for public health^1,2^. NAFLD is the leading cause of liver damage and dysfunction in the western world and is strongly associated with obesity and insulin resistance^3^. Fatty liver could subsequently develop to malignancy and end-stage liver failure^4–6^. Currently, NAFLD is a leading indication for liver transplantation in the United States^7^. Mechanistic, preclinical, and clinical studies provided an initial view into NAFLD progression along with the process from NAFLD to NASH and fibrosis^8,9^. However, owing to the epidemics of NAFLD and the unclear mechanism of NAFLD progression, it is important to elucidate the underlying NAFLD mechanisms in detail.

Gene profiling experiments in cross-sectional studies have been useful in identifying factors involved in NAFLD progression^10–12^. Gene profiling studies identified novel targets of NAFLD and demonstrated the roles of specific metabolic and repair pathways in the disease^11^. In the present study, we carried out a comprehensive bioinformatics analysis between normal liver tissues and NAFLD/NASH tissues on the Gene-Cloud of Biotechnology Information (GCBI) bioinformatics platform. Based on the comprehensive bioinformatics analysis, we determined to identify key differentially expressed genes (DEGs), biological processes and pathways that are closely associated with NAFLD/NASH.

## Materials and Methods

### Gene expression omnibus datasets

The Gene Expression Omnibus (GEO) (https://www.ncbi.nlm.nih.gov/gds) is a public repository at the National Center of Biotechnology Information for storing high-throughput gene expression datasets. We selected potential GEO datasets according to the following inclusion criteria: 1) specimens had histological diagnosis; 2) human liver tissues diagnosed as hepatocyte steatosis for the experimental group; 3) normal liver tissues used as controls; 4) expression profiling by array and raw data had the CEL format; 5) performed on the GPL570 platform ([HGU133_Plus_2] Affymetrix Human Genome U133 Plus 2.0 Array) and Human Exon 1.0 ST v1 Array; and 6) supported by GCBI analysis laboratory. Datasets with specimens from other organisms, expression profiling by RT-PCR (or genome variation profiling by SNP array/SNP genotyping by SNP array), analyses on platforms other than GPL570, or sample size <10 were excluded.

We used the search terms “Fatty liver” [MeSH Terms] AND “Homo sapiens” [Organism] and “Non-alcoholic” [Description] and “CEL” [Supplementary Files] and “Expression profiling by array” [DataSet Type] in the GEO DataSets to identify potential datasets. Then, we further screened these datasets according to the above inclusion criteria. Finally, 3 GEO datasets, GSE66676, GSE49541, and GSE83452, were included in our study.

### Gene-Cloud of Biotechnology Information (GCBI)

GCBI (Shanghai, China, https://www.gcbi.com.cn) is an online comprehensive bioinformatics analysis platform that combines a variety of research findings, genetic information, sample information, data algorithms and bioinformatics to create a “gene knowledge base,” which involves GEO datasets. GCBI platform can systematically analyze GEO dataset-derived gene expression information^13^, including more than 120 million copies of genomic samples. In the present study, GCBI was used to identify DEGs between NAFLD/NASH liver tissues and normal liver tissues. In the Differential Gene Expression Analysis module on the GCBI platform, we identified DEGs with a fold expression change >5 at cut off values Q < 0.05 and P < 0.05. Venn diagrams were used to compare the top 100 DEGs from 3 cohorts by Venny (http://bioinfogp.cnb.csic.es/tools/venny/index.html). Based on the DEGs, we further performed gene ontology (GO) analysis in terms of biological functions and Kyoto Encyclopedia of Genes and Genomes (KEGG) analysis in terms of pathway analysis. The top 20 biological functions and pathways are presented. Furthermore, a pathway relation network module was used to identify the core networks and pathway connections. Then, we applied the Gene Co-expression Network module on the GCBI platform to build gene co-expression networks for the DEGs to determine core genes in the networks.

### Tissue specimens, RNA extraction and qRT-PCR analysis

15 healthy liver tissues and 10 fatty liver tissues from liver donors were enrolled in our study to validate the expression levels of co-expressed DEGs. Prior patient consent and ethical approval from the ethics committee of the First Affiliated Hospital, Sun Yat-sen were obtained. All methods were performed in accordance with the ethics guidelines and regulations. We selected 8 co-expressed DEGs, including CD24, PZP, COL1A1, COL1A2, LUM, VCAN, THBS2 and EPHA3, for validation. All tissues were histologically diagnosed. Total RNA from the tissue specimens was isolated using TRIzol reagent (Invitrogen, Carlsbad, California, USA), and qRT-PCR was performed with SYBR® Green dye (TaKaRa, Shiga, Japan), following the manufacturer’s instructions. The primer sequences are provided in Supplementary Table S1. β-tubulin was used as a reference gene.

### Ethics statement

The research protocol was reviewed and approved by the Research Ethics Committee of the First Affiliated Hospital, Sun Yat-sen. All experiments were conducted in accordance with approved guidelines of the First Affiliated Hospital, Sun Yat-sen University.

### Ethical approval

All procedures performed in studies involving human participants were in accordance with the ethical standards of the ethical committee of the First Affiliated Hospital, Sun Yat-sen University were obtained and with the 1964 Helsinki Declaration and its later amendments or comparable. NO tissues were procured from prisoners. All the livers were procured in Organ Transplant Center, The First Affiliated Hospital, Sun Yat-sen University.

### Ethical standards

Informed consent was obtained from all individual participants included in the study.

### Statistical analysis

Data are presented as the mean ± SD for continuous variables. Student’s t-test and analysis of variance were used to evaluate significant differences in demographic data. All P values were two-sided, and P < 0.05 was defined as statistically significant. Analyses were carried out by the Statistical Package for the Social Science (SPSS) 22.0 (IBM, USA).

## Results

### Study design

**Figure 1:**
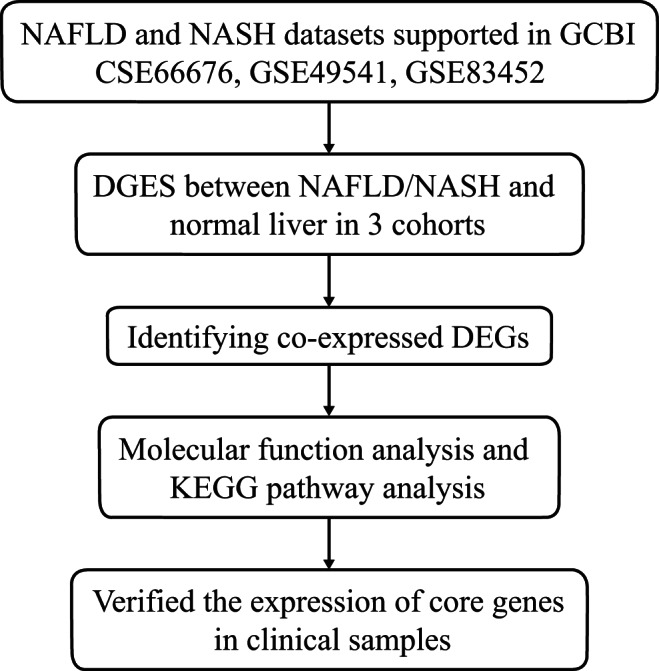
Flow diagram of the study design. NAFLD, nonalcoholic fatty liver disease; NASH, nonalcoholic steatohepatitis; DEGs, differentially expressed genes; KEGG, Kyoto Encyclopedia of Genes and Genomes.

The flow diagram of our study design is shown in Fig. 1. Our initial aim was to identify core genes in the development of fatty liver. We used 3 GEO datasets (GSE66676, GSE49541, and GSE83452) in the GCBI bioinformatics analysis platform. We extracted gene expression data of NAFLD/NASH and normal liver tissues to identify DEGs between the two histological diagnosis types in these 3 cohorts. Co-expressed DEGs were identified based on these DEGs. Biological function and KEGG pathway analyses were then performed. Finally, we verified expression of the core co-expressed genes in clinical samples to confirm the results.

### Major characteristics of samples in 3 datasets

GEO datasets GSE66676 (Cohort 1), GSE49541 (Cohort 2), and GSE83452 (Cohort 3) were enrolled in our study. All 3 datasets were available in the GCBI bioinformatics analysis platform. GSE66676 contained 33 NAFLD or NASH tissues and 34 normal liver tissues. GSE49541 contained 32 advanced NAFLD tissues and 40 mild NAFLD tissues. GSE83452 included 126 NASH tissues and 98 normal liver tissues.

### DEGs between NAFLD/NASH and normal liver tissues

**Figure 2:**
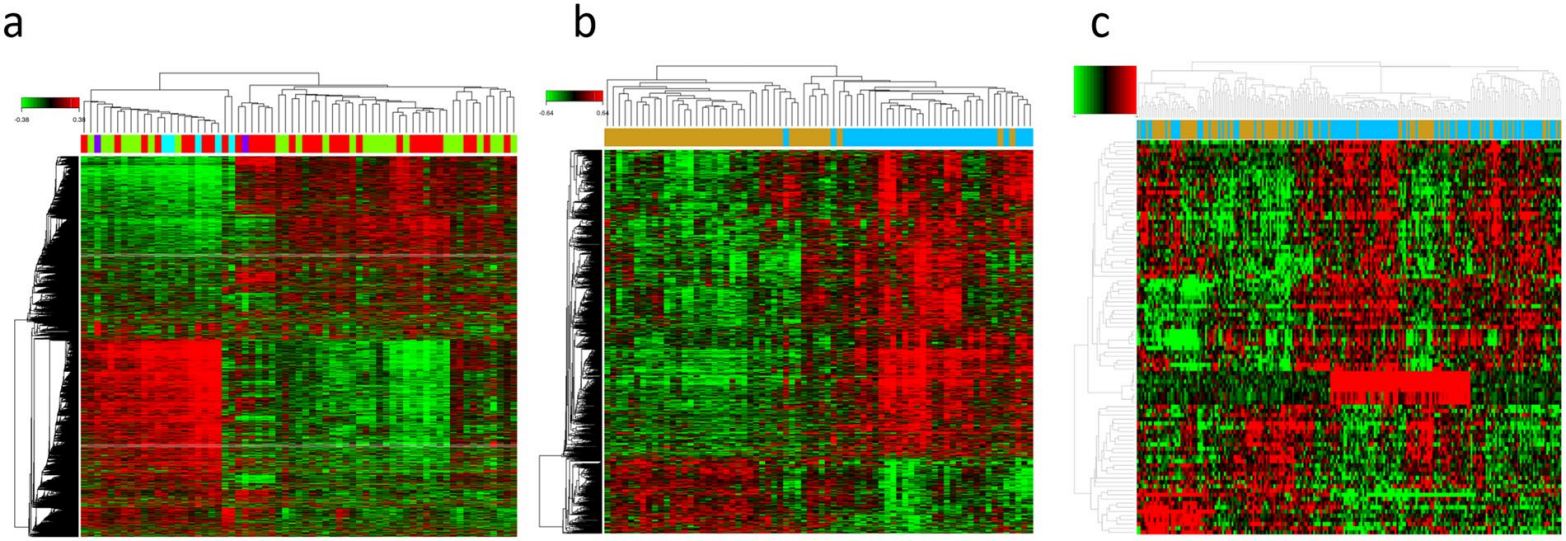
(**a–c**) Heat maps for potential DEGs between fatty liver and normal liver tissues in 3 cohorts. Heat maps for potential DEGs in GSE66676 (containing 33 NAFLD/NASH tissues and 34 normal liver tissues) (**a**), GSE49541 (containing contained 32 advanced NAFLD tissues and 40 mild NAFLD tissues) (**b**), and GSE83452 (126 NASH tissues and 98 normal liver tissues) (**c**).

**Figure 3:**
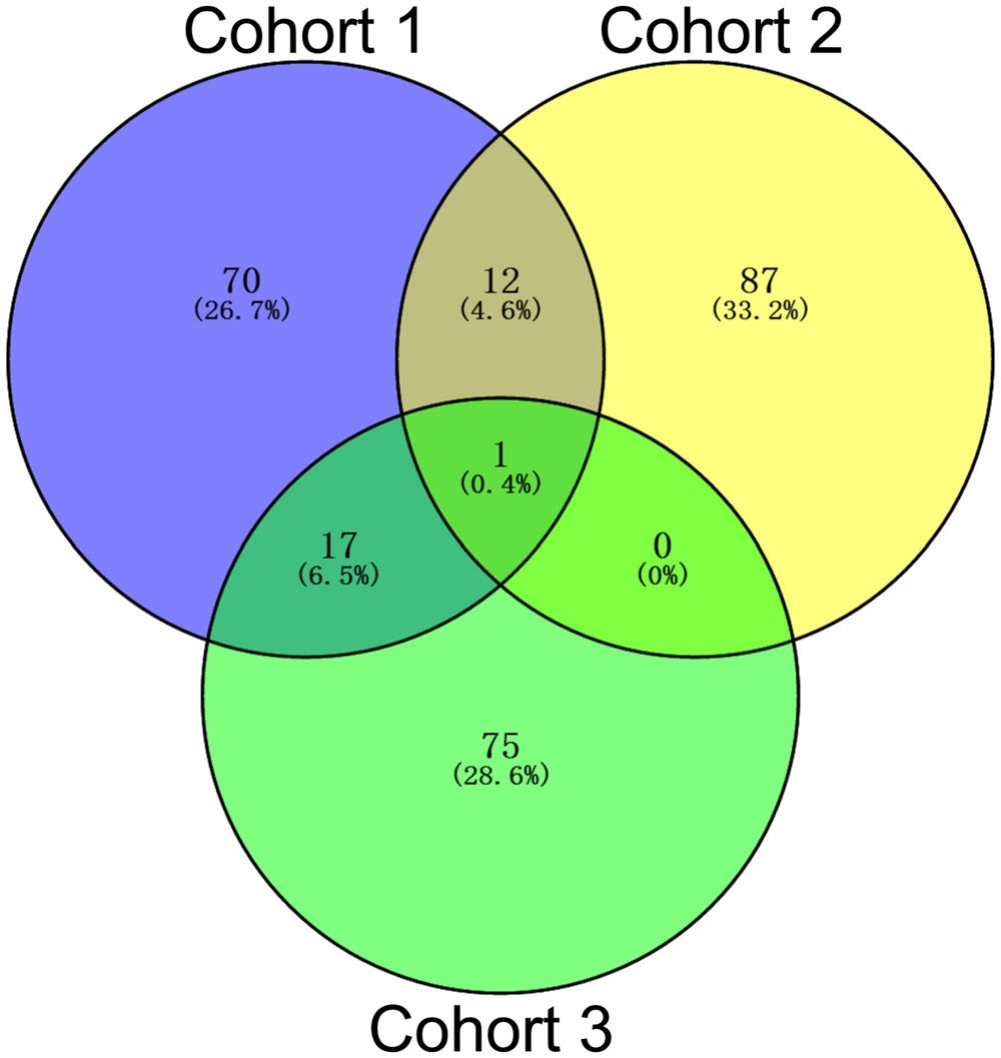
The Venn diagram shows the top 100 DEGs and co-expressed genes among cohort 1 (GSE66676), cohort 2 (GSE49541) and cohort 3 (GSE83452).

We identified 8503, 1538, and 94 potential DEGs in GSE66676, GSE49541, and GSE83452, respectively (Fig. 2a–c). The top 10 DEGs from 3 cohorts are shown in Tables 1–3. After removing duplicate genes and expression values lacking specific gene symbols, we used the top 100 DEGs from GSE66676 and GSE49541 and 93 DEGs from GSE83452 to create a Venn diagram. The intersection of these 3 datasets in Fig. 3 shows that CD24 was the only co-expressed DEG found in all 3 cohorts. Twelve genes were co-expressed in Cohort 1 and Cohort 2, including COL1A1, COL1A2, MOXD1, LUM, VCAN, EFEMP1, THBS2, MGP, COL3A1, EPHA3, BICC1, and COL6A3, all of which were upregulated. Seventeen genes were co-expressed in Cohort 1 and Cohort 3, including SPP1, FABP4, PZP, UTY, SLC1A2, GPNMB, DDX3Y, SAA1, MT1M, FAT1, USP9Y, LYZ, CXCL10, VIL1, EIF1AY, UBD, and CYP3A43. Further investigation showed that the regulation of MT1M, SLC1A2, CYP3A43 and VIL1 was not consistent. Therefore, 13 genes were co-expressed in Cohort 1 and Cohort 3.

**Table 1:** Top 10 differentially expressed genes in Cohort 1.

Rank	Probe Set ID	Gene Symbol	Gene Description	Regulation
1	7903765	GSTM1	glutathione S-transferase mu 1	Downregulated
2	8096301	SPP1	secreted phosphoprotein 1	Upregulated
3	8151532	FABP4	fatty acid binding protein 4	Upregulated
4	7960984	PZP	pregnancy-zone protein	Downregulated
5	7914000	NR0B2	nuclear receptor subfamily 0, group B, member 2	Downregulated
6	8174474	ACSL4	acyl-CoA synthetase long-chain family member 4	Upregulated
7	7908481	CFHR3	complement factor H-related 3	Upregulated
8	8136336	AKR1B10	aldo-keto reductase family 1, member B10	Upregulated
9	8150920	CYP7A1	cytochrome P450, family 7, subfamily A, polypeptide 1	Upregulated
10	7948420	FABP5	fatty acid binding protein 5	Upregulated

**Table 2:** Top 10 differentially expressed genes in Cohort 2.

Rank	Probe Set ID	Gene Symbol	Gene Description	Regulation
1	201842_s_at	EFEMP1	EGF containing fibulin-like extracellular matrix protein 1	Upregulation
2	201843_s_at	EFEMP1	EGF containing fibulin-like extracellular matrix protein 1	Upregulation
3	205422_s_at	ITGBL1	integrin, beta-like 1	Upregulation
4	201744_s_at	LUM	lumican	Upregulation
5	214247_s_at	DKK3	dickkopf WNT signaling pathway inhibitor 3	Upregulation
6	213071_at	DPT	dermatopontin	Upregulation
7	1557080_s_at	ITGBL1	integrin, beta-like 1 (with EGF-like repeat domains)	Upregulation
8	206070_s_at	EPHA3	EPH receptor A3	Upregulation
9	207173_x_at	CDH11	cadherin 11, type 2, OB-cadherin	Upregulation
10	209291_at	ID4	inhibitor of DNA binding 4	Upregulation

**Table 3:** Top 10 differentially expressed genes in Cohort 3.

Rank	Probe Set ID	Gene Symbol	Gene Description	Regulation
1	16829985	ENO3	enolase 3 (beta, muscle)	Upregulation
2	16766132	APOF	apolipoprotein F	Downregulation
3	16895179	TP53I3	tumor protein p53 inducible protein 3	Upregulation
4	17104259	MSN	moesin (MSN)	Upregulation
5	16789484	ADSSL1	adenylosuccinate synthase like 1 (ADSSL1)	Upregulation
6	16977052	CXCL10	chemokine (C-X-C motif) ligand 10	Upregulation
7	16890891	VIL1	villin 1	Downregulation
8	16909401	SLC16A14	solute carrier family 16, member 14	Upregulation
9	16811975	TSPAN3	tetraspanin 3	Upregulation
10	16841768	CENPV	centromere protein V	Downregulation

### Biological process analysis

**Figure 4:**
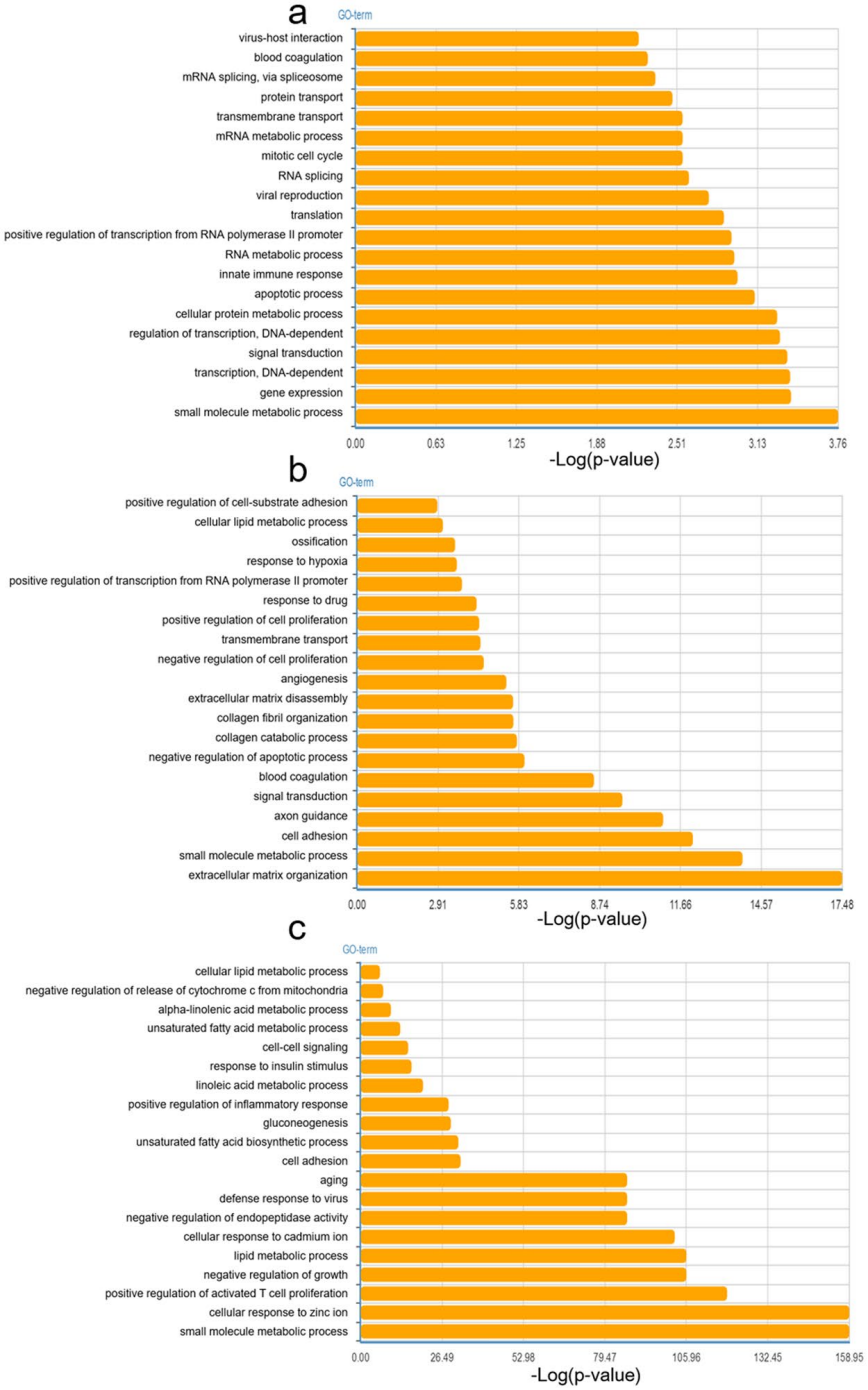
(**a–c**) Top 20 biological functions in terms of GO analysis related to DEGs in cohort 1 (**a**), cohort 2 (**b**) and cohort 3 (**c**).

In the present study, GO analysis was applied to investigate the biological function of the DEGs. The biological process analysis (Fig. 4a–c) revealed that “small molecule metabolic process” was a common biological function in 3 cohorts. “Cellular lipid metabolic process” and “cell adhesion” were the common biological processes in Cohort 2 and Cohort 3. Four common biological processes in Cohort 1 and Cohort 2 were “blood coagulation, transmembrane transport, positive regulation of transcription from RNA polymerase II promoter and signal transduction”. Metabolic processes, such as “alpha-linolenic acid metabolic process, unsaturated fatty acid metabolic process, mRNA/RNA metabolic process” and other biological processes, were also vital in each cohort.

### Pathway analysis

**Figure 5:**
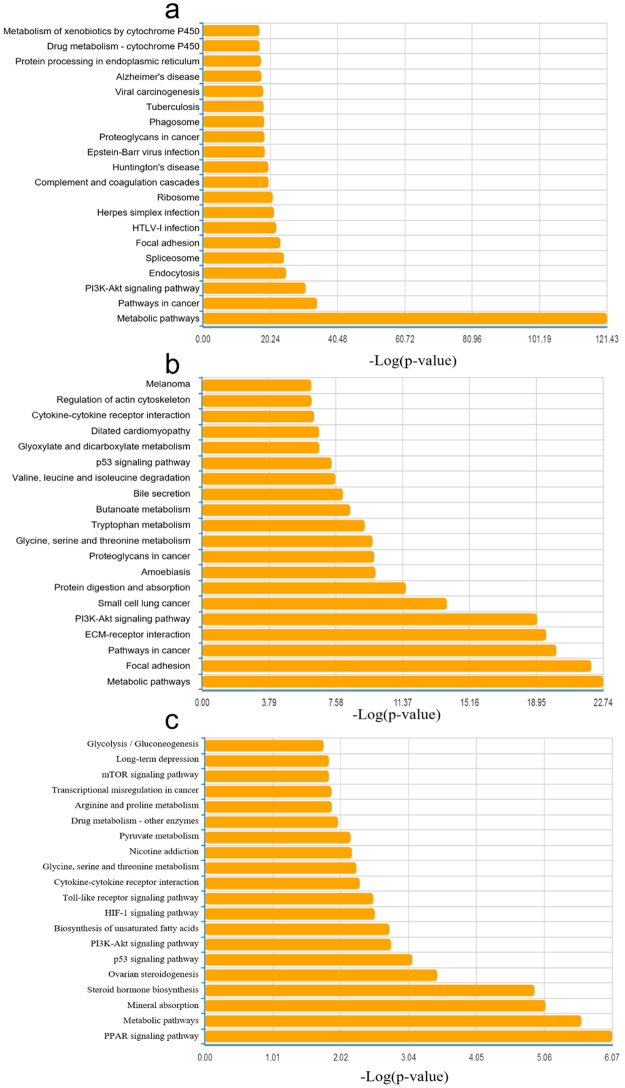
(**a–c**) Top 20 pathways in terms of KEGG pathway analysis related to DEGs in cohort 1 (**a**), cohort 2 (**b**) and cohort 3 (**c**). (**d–f**) Pathway relation network in 3 cohorts.

KEGG pathway analysis were used to investigate the pathway based on the DEGs identified. Figure 5a–c shows the top 20 pathways involved in each cohort. Among them, glycine, serine and threonine metabolism; cytokine-cytokine receptor interaction; PI3K-Akt signaling pathway; p53 signaling pathway; and metabolic pathways were 5 common pathways related to the fatty liver process. Among the top ten pathways in the pathway relation network in 3 cohorts, “glycolysis/gluconeogenesis”, “p53 signaling pathway” and “glycine, serine and threonine metabolism” were 3 intersecting pathways in the relation network (Table 4). MAPK signaling pathway, apoptosis, pathways in cancer, cell cycle, Wnt signaling pathway and pyruvate metabolism were the other intersecting pathways in pairwise comparisons.

**Table 4:** Top 10 pathways in pathway relation network of the 3 cohorts.

Rank	Cohort 1	Cohort 2	Cohort 3
1	MAPK signaling pathway	MAPK signaling pathway	Pyruvate metabolism
2	Apoptosis	Pathways in cancer	Glycolysis/Gluconeogenesis
3	Pathways in cancer	Apoptosis	Fatty acid biosynthesis
4	Cell cycle	Cell cycle	Glycine, serine and threonine metabolism
5	Glycolysis/Gluconeogenesis	p53 signaling pathway	Prostate cancer
6	p53 signaling pathway	Pyruvate metabolism	Cytokine-cytokine receptor interaction
7	Glycine, serine and threonine metabolism	Glycolysis/Gluconeogenesis	Toll-like receptor signaling pathway
8	Calcium signaling pathway	Wnt signaling pathway	p53 signaling pathway
9	Citrate cycle (TCA cycle)	Focal adhesion	
10	Wnt signaling pathway	Glycine, serine and threonine metabolism	

### The validation of core genes expression in clinical samples

**Figure 6:**
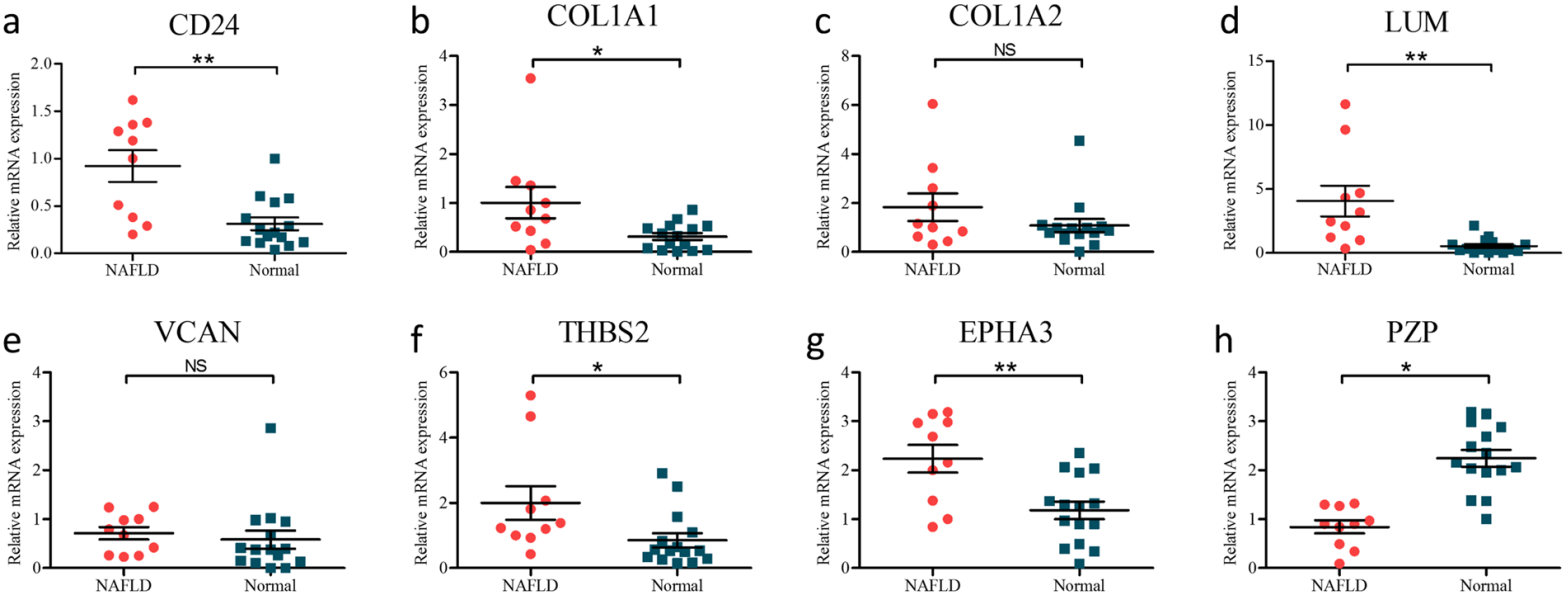
(**a–h**) Real-time qPCR validation of 8 co-expressed DEGs in 10 NAFLD and 15 normal liver tissues. *P < 0.05, **P < 0.01, NS represents no significant difference, analysis by non-paired t tests.

To further determine which genes might play a significant role in the progression of fatty liver, we used real-time qPCR to detect the expression of 8 DEGs using clinical samples, including CD24, PZP, COL1A1, COL1A2, LUM, VCAN, THBS2 and EPHA3. 15 healthy liver tissues and 10 fatty liver tissues from liver donors were enrolled. We noted that CD24, COL1A1, LUM, THBS2 and EPHA3 were usually upregulated in fatty liver tissues comparing to normal liver tissues, which is consistent with the results of bioinformatics analysis above (Fig. 6a–f). PZP was significantly downregulated in fatty livers compared with normal livers, which is also consistent with the co-expressed results (Fig. 6h).

## Discussion

Non-alcoholic steatohepatitis is strongly associated with strong genetic component and dietary component^1^. A number of genes could be associated with the susceptibility and development of NAFLD and NASH^1,2^. In our present study, we imported three GEO datasets into the GCBI comprehensive analysis platform to extract gene expression data of NAFLD/NASH tissue comparing to normal liver tissue or NASH tissue comparing to NAFLD liver tissue. We identified co-expressed DEGs, common biological processes and pathways between NAFLD/NASH and normal liver tissues through differential expression analysis in GCBI.

We found that CD24, PZP, COL1A1, COL1A2, LUM, VCAN, THBS2 and EPHA3 were potential biomarkers for distinguishing NAFLD/NASH. Further validation by clinical samples, the expression of CD24, PZP, COL1A1, LUM, THBS2 and EPHA3 were significantly regulated. Moreover, CD24 is a core gene among these DEGs and have not yet been studied of its impact on hepatocyte steatosis. CD24 encodes a sialoglycoprotein that is expressed on mature granulocytes and B cells and modulates growth and differentiation signals to these cells^13^. Several studies have reported that CD24 is widely distributed, including on hematopoietic cells^14^ and non-hematopoietic cells^15,16^. CD24 has been studied to be associated with various pathophysiological processes, including tracking divergent states of cells^17^, regulating CD8 (+) T cell activation^18^ and participating in mutant-IDH1-dependent chromatin state reprogramming^19^. Accumulating evidence showed that this protein is overexpressed in many types of cancers, resulting in cancer cell growth, proliferation and metastasis^20^. Previous studies have reported that CD24 is overexpressed in nearly 70% of human cancers, and intracellular CD24 disrupts the ARF-NPM interaction and enables mutational and viral oncogene-mediated p53 inactivation^21^. CD24 is significantly correlated with tumorigenesis genes, such as non-coding RNAs^22^. It was previously demonstrated that a subpopulation of adipocyte progenitor cells was identified with the expression of the cell surface molecule CD24 being critically important for reconstitution of white adipose tissue function *in vivo*^23^. CD24 was shown to be important in the reconstitution of white adipocyte (WAT) function *in vivo*, as well as a specific regulator of adipogenesis *in vitro*^23,24^. Furthermore, it was demonstrated that losing CD24 in male mice leads to a generalized reduction of WAT and metabolic disturbances^24^. Fairbridge *et al*.^25^. reported that the global absence of CD24 affects adipocyte cell size *in vivo* in a sex- and diet-dependent manner, as well as causing metabolic disturbances in glucose homeostasis and free fatty acid levels. However, the precise function of CD24 and the underlying mechanisms of its activity in NAFLD/NASH progression remain unclear. This is the first study to identify the prominent correlation between CD24 and NAFLD/NASH.

Insulin resistance (IR) is central in the pathogenesis of NAFLD. NAFLD often significantly impacts glucose and lipid metabolism by exacerbating hepatic IR^26^. CD24 can regulate lipid raft occupancy and may affect glucose uptake by regulating lipid raft protein localization^27^. Additional studies will be required to identify whether there is a potential mechanism among CD24, glucose uptake, and insulin resistance. IR is a major factor for hepatic fat accumulation^28^. Several studies only focused on a single protein, which ignored the various potential pathways in this complex disease^29^. In our study, we found that “glycolysis/gluconeogenesis”, “p53 signaling pathway” and “glycine, serine and threonine metabolism” were 3 intersecting pathways in the relation network. Several studies have reported that glycolysis/gluconeogenesis^30^, the p53 signaling pathway^31^, and glycine metabolism^32^ were each associated with IR. The biological process “small molecule metabolic process” was reported to be vital in cancer^33^, hepatic metabolism disorder like IR^34^. We supposed it to be a key biological process in fatty liver. Further studies will be required to determine whether the co-expressed DEGs including CD24 could activate one of these pathways or other mechanisms to induce NAFLD/NASH in humans.

In summary, we first used the GCBI bioinformatics analysis platform to identify DEGs between NAFLD/NASH tissues and normal liver tissues, which showed that CD24 is the hub gene and identify 3 intersecting pathways in the relation network. Then, using qRT-PCR analysis, we concluded that the mRNA expression of CD24 is upregulated in fatty liver. Further studies are required to elucidate the function and underlining mechanisms of this potential biomarker in the progression of hepatocyte steatosis.

### Electronic supplementary material

Table S1
