# Author Correction: A comprehensive bioinformatics analysis on multiple Gene Expression Omnibus datasets of nonalcoholic fatty liver disease and nonalcoholic steatohepatitis

**DOI:** 10.1038/s41598-019-39022-7

**Published:** 2019-05-03

**Authors:** Shanzhou Huang, Chengjun Sun, Yuchen Hou, Yunhua Tang, Zebin Zhu, Zhiheng Zhang, Yixi Zhang, Linhe Wang, Qiang Zhao, Mao-Gen Chen, Zhiyong Guo, Dongping Wang, Weiqiang Ju, Qi Zhou, Linwei Wu, Xiaoshun He

**Affiliations:** 1 Organ Transplant Center, The First Affiliated Hospital, Sun Yat-sen University, Guangzhou, 510080 China; 2 Guangdong Provincial Key Laboratory of Organ Donation and Transplant Immunology, Guangzhou, 510080 China; 3 Guangdong Provincial International Cooperation Base of Science and Technology (Organ Transplantation), Guangzhou, 510080 China; 4 Department of General Surgery, Hui Ya Hospital of The First Affiliated Hospital, Sun Yat-sen University, Huizhou, Guangdong, 516081 China

Correction to: *Scientific Reports* 10.1038/s41598-018-25658-4, published online 16 May 2018

This Article contained errors in the accession codes provided for the data used in the study, and in the description of Methods.

The correct accession codes are as follows: Cohort 1 data is available in GEO under accession GSE66676 and Cohort 3 is available under accession GSE83452. As a result, in all instances in the Article where the accession numbers were being referenced GSE31803 was replaced with GSE66676 and GSE63067 was replaced with GSE83452.

Additionally, in Materials and Methods, under the subheading “Gene expression omnibus datasets”:

“We selected potential GEO datasets according to the following inclusion criteria: 1) specimens had histological diagnosis; 2) human liver tissues diagnosed as hepatocyte steatosis for the experimental group; 3) normal liver tissues used as controls; 4) expression profiling by array and raw data had the CEL format; 5) performed on the GPL570 platform ([HGU133_Plus_2] Affymetrix Human Genome U133 Plus 2.0 Array); and 6) supported by GCBI analysis laboratory”.

now reads:

“We selected potential GEO datasets according to the following inclusion criteria: 1) specimens had histological diagnosis; 2) human liver tissues diagnosed as hepatocyte steatosis for the experimental group; 3) normal liver tissues used as controls; 4) expression profiling by array and raw data had the CEL format; 5) performed on the GPL570 platform ([HGU133_Plus_2] Affymetrix Human Genome U133 Plus 2.0 Array) and Human Exon 1.0 ST v1 Array; and 6) supported by GCBI analysis laboratory”.

Figure 1 is replaced to correct the accession codes in the flowchart.

In the Results section, under the subheading “Major characteristics of samples in 3 datasets”:

“GEO datasets GSE31803 (Cohort 1), GSE49541 (Cohort 2), and GSE63067 (Cohort 3) were enrolled in our study. All 3 datasets were available in the GCBI bioinformatics analysis platform. GSE31803 contained 33 NAFLD or NASH tissues and 34 normal liver tissues. GSE49541 contained 32 NAFLD tissues and 40 normal liver tissues. GSE63067 included 126 NASH tissues and 98 normal liver tissues”.

now reads:

“GEO datasets GSE66676 (Cohort 1), GSE49541 (Cohort 2), and GSE83452 (Cohort 3) were enrolled in our study. All 3 datasets were available in the GCBI bioinformatics analysis platform. GSE66676 contained 33 NAFLD or NASH tissues and 34 normal liver tissues. GSE49541 contained 32 advanced NAFLD tissues and 40 mild NAFLD tissues. GSE83452 included 126 NASH tissues and 98 normal liver tissues.”

In Figure 2 legend:

“Heat maps for potential DEGs in GSE31803 (containing 33 NAFLD/NASH tissues and 34 normal liver tissues) (**a**), GSE49541 (containing 32 NAFLD tissues and 40 normal liver tissues) (**b**), and GSE63067 (126 NASH tissues and 98 normal liver tissues) (**c**)”.

now reads:

“Heat maps for potential DEGs in GSE66676 (containing 33 NAFLD/NASH tissues and 34 normal liver tissues) (**a**), GSE49541 (containing contained 32 advanced NAFLD tissues and 40 mild NAFLD tissues) (**b**), and GSE83452 (126 NASH tissues and 98 normal liver tissues) (**c**)”.

In the Discussion section:

“In our present study, we imported three GEO datasets into the GCBI comprehensive analysis platform to extract NAFLD/NASH tissue and normal liver tissue gene expression data”.

now reads:

“In our present study, we imported three GEO datasets into the GCBI comprehensive analysis platform to extract gene expression data of NAFLD/NASH tissue comparing to normal liver tissue or NASH tissue comparing to NAFLD liver tissue”.

The conclusions of the Article are unaffected by these changes.

