# Supplementary material for: A comprehensive bioinformatics analysis on multiple Gene Expression Omnibus datasets of nonalcoholic fatty liver disease and nonalcoholic steatohepatitis
Source: Sci Rep. 2018 May 16;8:7630. doi: 10.1038/s41598-018-25658-4 (PMC5955936; doi:10.1038/s41598-018-25658-4)
Supplement: Supplementary file 1 — Table S1 [file 41598_2018_25658_MOESM1_ESM.pdf]

A comprehensive bioinformatics analysis on multiple Gene Expression Omnibus datasets of nonalcoholic fatty liver disease and nonalcoholic steatohepatitis

Shanzhou Huang[1,2,3,†], Chengjun Sun[1,2,3,†], Yuchen Hou[1,2,3,†], Yunhua Tang[1,2,3], Zebin Zhu[1,2,3], Zhiheng Zhang[1,2,3], Yixi Zhang[1,2,3], Linhe Wang[1,2,3], Qiang Zhao[1,2,3], Mao-Gen Chen[1,2,3], Zhiyong Guo[1,2,3], Dongping Wang[1,2,3], Weiqiang Ju[1,2,3], Qi Zhou[4,*], Linwei Wu[1,2,3,*], Xiaoshun He[1,2,3,*]

[1]Organ Transplant Center, The First Affiliated Hospital, Sun Yat-sen University, Guangzhou 510080, China

[2]Guangdong Provincial Key Laboratory of Organ Donation and Transplant Immunology, Guangzhou 510080, China

[3]Guangdong Provincial International Cooperation Base of Science and Technology (Organ Transplantation), Guangzhou 510080, China

[4] Department of General Surgery, Hui Ya Hospital of The First Affiliated Hospital, Sun Yat-sen University, Huizhou, Guangdong 516081, China.

***Corresponding authors:** Xiaoshun He, M.D., Linwei Wu, M.D., Qi Zhou, M.D., Organ Transplant Center, The First Affiliated Hospital, Sun Yat-sen University, NO. 58 Zhongshan Er Road, Guangzhou 510080, China (E-mail: gdtrc@163.com, lw97002@163.com and hnzhouqi@163.com)

†These authors contributed equally to the article and should be considered co-first authors.

Table S1: Sequence of primers used for validation of expression levels of co-expressed DEGs.

Genes	Sequence
CD24-S	TGCTCCTACCCACGCAGATT
CD24-A	TTGGTGGTGGCATTAGTTGGAT
COL1A1-S	GACATCCCACCAATCACCTGC
COL1A1-A	TCGACGCCGGTGGTTTCTT
COL1A2-S	TGCCTAGCAACATGCCAATCTT
COL1A2-A	TGAGCAGCAAAGTTCCCACC
EPHA3-S	AACTGATTCCGCAGCCTTCC
EPHA3-A	GGACCCAGTTTGTTCTCAGCC
LUM-S	CTGGCTGATAGTGGAATACCTGG
LUM-A	GGTTTCTGAGATGCGATTGCC
THBS2-S	TCTGGACGAGCCCTTCTACGA
THBS2-A	GATGGCGTTGATCTCAGCTCC
TUBB-S	CTGACCACACCAACCTACGG
TUBB-A	CCAGGCATAAAGAAATGGAGAC
VCAN-S	TCAGAAGGCTTGTTTGGACGTT
VCAN-A	CAGCCTCCTCGAAGGTGAATT
PZP-A	TGGCAGTTTGGTTGGTCGTAG
PZP-S	CAGCAAAGACAGTCAGCACCAC

-S, sense; -A, anti-sense.
